# Two New Neolignans from *Syringa velutina* Kom

**DOI:** 10.3390/molecules14030953

**Published:** 2009-02-27

**Authors:** Xue-Song Feng, Yang Qu, Lei Xu, Da-Cheng Wang, Li-Jun Wu, Fan-Hao Meng, Ya-Ru Liu

**Affiliations:** 1 School of Pharmaceutical Sciences, China Medical University, Shenyang, Liaoning, 110001, P. R. China; E-mails: fhmeng@cmu.edu.cn (F-H.H.), free1962514@yahoo.com.cn (Y-R.L.); 2 School of Traditional Chinese Medicines, Shenyang Pharmaceutical University, Shenyang, Liaoning, 110016, P. R. China; E-mails: quyang_7@yahoo.com.cn (Y.Q.), abcgdef6001@163.com (L.X.) , wulijun_111@hotmail.com (L-J.W.); 3 College of Animal Science and Veterinary Medicine, Jilin University, Changchun, Jilin, 130062, P. R. China; E-mail: wdc9928@yahoo.com.cn (D-C.W.)

**Keywords:** *Syringa velutina* Kom., Neolignans.

## Abstract

Two new neolignans, (7*S*,8*R*)-guaiacylglycerol-8-*O*-4′-sinapyl ether 9′-*O*-β-D- glucopyranoside (**1**) and (7*S*,8*R*)-syringylglycerol-8-*O*-4′-sinapyl ether 9′-*O*-β-D-gluco- pyranoside (**2**) were isolated from the leaves of *Syringa velutina* Kom.. Their structures were established by chemical properties and spectroscopic evidence.

## Introduction

*Syringa* (*S*.) *velutina* Kom. has been widely cultivated in the northern parts of China and Korea. Its leaves, flower buds, and bark have been used for many centuries in traditional Chinese medicine to treat infectious fevers, counteract inflamations, dampness and acute icteric hepatitis [1]. A previous phytochemical study on the genus *Syringa* led to the isolation of lignans, iridoid glucosides and so on [2]. For the purpose to finding more bioactive agents, a study on chemical constituents of *S*. *velutina* was carried out, and two new 8-*O*-4′ neolignans (compounds **1** and **2**) along with four known lignans: i.e. (+)-syringaresinol-di-*O*-β-D-glucopyranoside (**3**), (+)-medioresinol-di- *O*-β-D-glucopyranoside (**4**), (-)-olivil (**5**), (–)secoisrariciresinol-9-*O*-β-D-glucopyranoside (**6**) [2,3,4] were obtained. In this paper, the isolation and structural elucidation of the two new compounds are reported.

## Results and Discussion

Compound **1** was obtained as a white amorphous powder (MeOH) with a negative optical rotation [α]^23^_D_ -9.1° (*c*=0.2, MeOH). The IR spectrum of **1** showed absorption bands at 3,321, 1,607, 1,500, 1,064, and 1,021 cm^-1^, ascribable to hydroxyl, aromatic and ether functions. The positive- and negative-ion HR-ESI-MS of **1** showed quasimolecular ion peaks at *m/z* 591.2062 [M+Na]^+^ and 567.2077 [M-H]^-^, which revealed the molecular formula of 1 to be C_27_H_36_O_13_. Acid hydrolysis of 1 with 1M HCl liberated D-glucose, which was identified by HPLC analysis using an optical rotation detector [5]. The ^1^H-NMR spectrum of 1 showed signals assignable to one 1,2,4-trisubstituted aromatic ring [δ 6.68 (1H, d, *J* = 8.1Hz), 6.71 (1H, dd, *J* = 8.1, 1.8 Hz), 6.90 (1H, d, *J* = 1.8 Hz)], one 1,3,4,5-tetrasubstituted aromatic ring [δ 6.73 (2H, s)], which indicated a partial symmetric structure for 1, one *trans*-double bond [δ 6.31 (1H, dt, *J* = 15.9, 5.4 Hz), 6.56 (1H, d, *J* = 15.9 Hz)], one anomeric proton [δ 4.20 (1H, d, *J* = 7.8 Hz)], three methoxy groups [δ 3.72 (3H, s), 3.75 (6H, s)], along with other alkyl groups signals. The ^13^C-NMR spectrum showed 27 carbon signals, including one 1,2,4- trisubstituted aromatic ring [δ 133.5, 111.1, 147.2, 145.5, 114.9, 119.6], one 1,3,4,5-tetrasubstituted aromatic ring [δ 132.1, 104.0, 153.0, 135.4, 153.0, 104.0], two olefinic carbons [δ 125.9, 131.5], one glucose unit [δ 102.3, 73.7, 77.1, 70.3, 76.9, 61.3], three methyoxyl carbons [δ 55.7, 56.2, 56.2], and four other alkyl carbon signals, the chemical shifts of which indicated the connection to an oxygen atom. The direct correlations between the proton and carbon were assigned by its HSQC spectrum (Table 1). According to the spectral data above, the structure of **1** was supposed to contain one guaiacylglycerol, one sinapyl alcohol [6] and one glucose moiety [7]. The glucose anomeric configuration was assigned to be *β* based on the *J*-value of its anomeric proton. Furthermore, the HMBC correlations between δ 4.11 (H-8) and δ 135.4 (C-4′), δ 4.20 (H-1″) and δ 68.8 (C-9′), connected the partial structures above together. Irritating δ 4.77 (H-7), δ 4.11 (H-8) showed NOE correlations in the difference Nuclear Overhauser Effect (dNOE) spectrum, which indicated that **1** possessed an *erythro* relative configuration. The relative structure was further confirmed by NOESY correlations between H-8/H-2, 7, 6, and H-9(δ 4.40)/H-2, 6. Enzymatic hydrolysis of **1** with cellulase gave the aglycone **1a**. The *erythro* configuration was also confirmed by the *J*_7, 8_-value (3.8 Hz) of **1a** [8]. The absolute structure of **1** was determined to be (7*S*, 8*R*) based on the negative cotton effects of **1** and **1a** in its CD spectrum [8]. The results above identified the structure of **1** as (7*S*,8*R*) guaiacylglycerol-8-*O*-4′-sinapyl ether 9′-*O*-*β*-D-glucopyranoside. 

**Figure 1:**
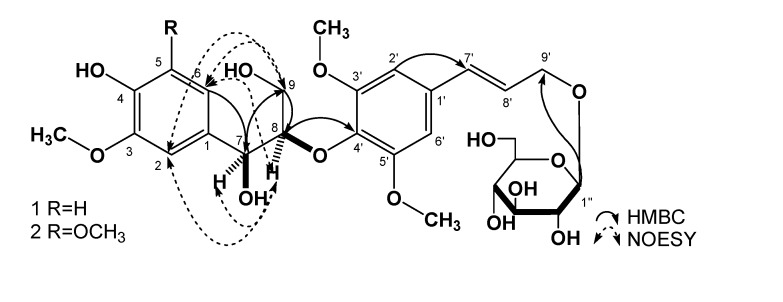
Structures and key HMBC, NOESY correlations of compound **1**, **2**.

**Table 1:** ^13^C-NMR (150 MHz), ^1^H-NMR (600 MHz) spectral data of compound **1**, **2** and HMBC correlations of **1** (in DMSO-*d*_6_, *δ* ppm, *J* Hz).

position	1			2	
	*δ*_C_	*δ*_H_	HMBC(H→C)	*δ*_C_	*δ*_H_
1	133.5			132.6	
2	111.1	6.90 (1H, d, *J* = 1.8 Hz)	C-1, 3, 4, 6, 7	104.4	6.60 (1H, s)
3	147.2			147.6	
4	145.5			134.4	
5	114.9	6.68 (1H, d, *J* = 8.1 Hz)	C-1, 3, 4, 6	147.6	
6	119.6	6.71 (1H, dd, *J* =8.1, 1.8 Hz)	C-1, 2, 4, 5, 7	104.4	6.60 (1H, s)
7	72.2	4.77 (1H, dd, *J* =4.8, 4.8 Hz)	C-1, 2, 6, 8, 9	72.4	4.81 (1H, dd, *J* =4.8, 4.8 Hz)
8	86.4	4.11 (1H, m)	C-4′	86.4	4.15 (1H, m)
9	59.9	3.39 (1H, m)3.69 (1H, m)	C-7, 8	59.9	3.40 (1H, m)3.68 (1H, m)
1′	132.1			132.0	
2′, 6′	104.0	6.73 (2H, s)	C-1′, 4′, 7′	103.9	6.75 (2H, s)
3′, 5′	153.0			152.8	
4′	135.4			135.5	
7′	131.5	6.56 (1H, d, *J* = 15.9 Hz )	C-1′, 8′, 9′	131.4	6.57 (1H, d, *J* = 15.6 Hz )
8′	125.9	6.31 (1H, dt, *J* = 15.9, 5.4 Hz )	C-1′, 7′, 9′	125.8	6.34 (1H, dt, *J* = 15.6, 5.4 Hz )
9′	68.8	4.19 (1H, dd, *J* = 14.4, 5.4 Hz)4.40 (1H, dd, *J* = 14.4, 5.4 Hz)	C-7′, 8′	68.7	4.19 (1H, dd, *J* = 14.4, 5.4 Hz)4.41 (1H, dd, *J* = 14.4, 5.4 Hz)
4-OH		8.08 (1H, s)	C-3, 4, 5		
7-OH		5.10 (1H, d, *J* = 4.8 Hz)	C-1, 7, 8		
9-OH		4.03 (1H, t, *J* = 6.0 Hz)	C-9		
Glucose					
1′′	102.3	4.20 (1H, d, *J* = 7.8 Hz)	C-9′	102.2	4.21 (1H, d, *J* = 7.8 Hz)
2′′	73.7	2.99 (1H, td, *J* = 7.8, 4.8 Hz)		73.6	3.05(1H,m)
3″	77.1	3.08 (1H, m)		77.0	3.09 (1H, m)
4′′	70.3	3.04 (1H, m)		70.2	3.07 (1H, m)
5′′	76.9	3.13 (1H, td, *J* = 9.0, 4.8 Hz)		76.9	3.14 (1H, m)
6′′	61.3	3.43 (1H, m)3.67 (1H, m)		61.2	3.44 (1H, dd, J = 12.0, 6.0 Hz)3.67 (1H, m)

Compound **2** was obtained as a white amorphous powder (MeOH) with a negative optical rotation [α]^23^_D_ -9.4° (*c*=0.1, MeOH). The IR spectrum of **2** showed absorption bands at 3,308, 1,647, 1,541, 1,489, 1,061, and 1,020 cm^-1^, ascribable to hydroxyl, aromatic and ether functions. The positive- and negative-ion HRESI-MS of **2** showed quasimolecuar ion peaks at *m/z* 621.2161 [M+Na]^+^ and 597.2182 [M-H]^-^, which revealed the molecular formula of **2** to be C_28_H_38_O_14_. Acid hydrolysis of **2** with 1 M HCl liberated D-glucose, which was identified by HPLC analysis using an optical rotation detector [5]. The ^1^H- and ^13^C-NMR spectral data of compound **2** were similar to those of **1**. By comparing the spectral data of **2** with those of **1**, a syringylglycerol moiety [δ 132.0, 104.4, 147.6, 131.5, 147.6, 104.4] was observed instead of the guaiacylglycerol unit in **1**. Irridiating δ 4.81 (H-7), δ 4.15 (H-8) showed a NOE correlation in the dNOE spectrum, which indicated that **2** also possessed an *erythro* relative configuration. Thus, the structure was identified as (7*S*,8*R*) syringylglycerol- 8-*O*-4′-sinapyl ether 9′-*O*-*β*-D-glucopyranoside, which has the same stereostructure as **1** based on the *J*-value of H-7 and negative Cotton effect in its CD spectrum .

## Experimental

### General

The following instruments were used to obtain physical data: specific rotations, Perkin-Elmer 241MC (*l* = 5 cm); UV spectra, Shimadzu UV-1700 spectrometer; IR spectra, Bruker IFS-55 spectrometer; high-resolution ESI-MS, LC-MSD-Trap-SL mass spectrometer; CD detector, JASCO CD-2095-plus; ^1^H- and ^13^C-NMR spectra, Bruker AV-600 (600 MHz) spectrometer, with tetramethylsilane as an internal standard; and HPLC instrument, Shimadzu LC-9A and SPD-M6A PAD (UV-VIS)detector; HPLC column, COSMOSIL 5C18-PAQ (250×4.6 mm i.d.) and YMC-PACK ODS-a (250×10 mm i.d.) columns were used for analytical and preparative purposes, respectively. The following experimental conditions were used for chromatography: D101 macroporous resins (TianJin Ou-Rui Bio-Tech Co. Ltd., Tianjin, P.R. China); ordinary-phase silica gel column chromatography, Silica gel (200–300 mesh, Qingdao Haiyang Chemical, co. Ltd., Qingdao, P.R. China); TLC Silica gel GF_254_ (normal phase, Qingdao Haiyang Chemical, Co. Ltd.); and detection was achieved by spraying with 1 % Ce(SO_4_)_2_ -10 % aqueous H_2_SO_4_, followed by heating.

### Plant material

The leaves of *S. velutina* Kom. were collected in Xinmin, Liaoning Province, China in May 2005 and identified by Prof. Qishi Sun (School of Traditional Chinese Medicine, Shenyang Pharmaceutical University). A voucher specimen (No. 20050517) was deposited in the Herbarium of Section of medicinal plants of Shenyang Pharmaceutical University.

### Extraction and Isolation

The air-dried material (5 kg) was extracted with 70% ethanol (20 L) at room temperature for two weeks. The extract was then filtered and concentrated under reduced pressure to afford a viscous mass (1,560 g, 31.2 %), which was suspended in water and then subjected to D101 macroporous resin chromatography [ethanol-water (0:100→30:70→50:50→70:30→100:0 v/v)]. Five fractions were collected: the water soluble fraction (723 g, 14.5%), 30% ethanol-water soluble fraction (450 g, 9.0%), 50% ethanol-water soluble fraction (228 g, 4.6%), 70% ethanol-water soluble fraction (48 g, 1.0%) and 100% ethanol soluble fraction (93 g, 1.9%). A part of the 30% ethanol-water soluble fraction (300 g) was subjected to column chromatography on silica gel (2000 g). The column was eluted with CHCl_3_-MeOH (100:0→50:1→30:1→10:1→0:100 v/v), and five fractions [Fr.1 (6 g), Fr.2 (44 g), Fr.3 (56 g), Fr. 4 (17 g), Fr. 5 (146 g)] were collected. Fraction 4 was further isolated by semi-prep. HPLC (MeOH:H_2_O 40:60) to give **1** (14.1 mg, 0.0003 %) and **2** (10.1 mg, 0.0002%), respectively. The compounds **1** and **2** were identified by [α]_D_, NMR, MS, CD data

*(7S,8R)-Guaiacylglycerol-8-O-4′-sinapyl ether 9′-O-β-D-glucopyranoside* (**1**). White amorphous powder (MeOH); [α]^23^_D_ -9.1° (*c*=0.2, MeOH); CD (MeOH) Δε -9.4 (236); IR (KBr) *ν*_max_ cm^–1 ^3,321, 1,607, 1,500, 1,064, 1,021; HRESI-MS *m/z* 591.2062 [M+Na]^+^ and 567.2077 [M-H]^-^ (calcd. for C_27_H_36_O_13_Na 591.2054 and C_27_H_35_O_13_ 567.2078); ^13^C- (150 MHz) and ^1^H-NMR (600 MHz) spectral data see Table 1.

*(7S,8R)-Syringylglycerol-8-O-4′-sinapyl ether 9′-O-β-D-glucopyranoside* (**2**). White amorphous powder (MeOH); [α]23D -9.4° (*c*=0.2, MeOH); CD (MeOH) Δε -9.8 (236); IR (KBr) *ν*_max_ cm^–1^ 3,308, 1,647, 1,541, 1,489, 1,061, 1,020; HRESI-MS *m/z* 621.2161 [M+Na]^+^ and 597.2182 [M-H]^-^ (calcd. for C_28_H_38_O_14_Na 621.2159 and C_28_H_37_O_14_ 597.2183);^ 13^C- (150 MHz), ^1^H-NMR (600 MHz) spectral data see Table 1.

### Acid hydrolysis of **1** and **2**

Compound **1** (4 mg) and **2** (2 mg) were refluxed with 1M HCl in 75% EtOH (2 mL) for 7 hrs, respectively. After cooling, the reaction mixture was neutralized with IRA-400 (OH^-^ form). Then, the filtrate was extracted with EtOAc. The aqueous layer was subjected to HPLC analysis under the following conditions: HPLC column, YMC-Pack NH_2_ (NH12S05-2546WT), 250×4.6 mm i.d. (YMC Co., Ltd, Japan); detection, optical rotation [Jasco OR-2090 Chiral Detector (Jasco Electric Co., Ltd., Japan)]; mobile phase, CH_3_CN-H_2_O (85:15, v/v); flow rate 1.0 mL/min; column temperature: room temperature. Identification of D-glucose from **1** and **2** present in the aqueous layer was carried out by comparison of its retention time and optical rotation with those of authentic sample. *t*_R_: 11.4 min (positive optical rotation).

### Enzymatic hydrolysis of **1**

An aqueous solution (8.0 ml) containing **1** (7.0 mg) and cellulase (36 mg) was incubated at 40°C for 4 d. The reaction mixture was extracted with EtOAc, and the EtOAc layer was evaporated under reduced pressure. The residue was purified by silica gel column chromatography [CHCl_3_-MeOH-H_2_O 30:3:1] to give (7*S*,8*R*) guaiacylglycerol-8-*O*-4′-sinapyl ether (**1a**, 2.5 mg). White amorphous powder (MeOH), [α]^23^_D_ +10.1° (*c*=0.1, MeOH). CD(MeOH) Δε -4.6 (236). ^1^H-NMR (CDCl_3_, 600 MHz) δ: 6.98 (1H, d, *J* = 1.8 Hz, H-2), 6.88 (1H, d, *J* = 8.2 Hz, H-5), 6.76 (1H, dd, *J* = 8.2, 1.8 Hz), 4.98 (1H, d, *J* = 3.8 Hz, H-7), 4.14 (1H, m, H-8), 3.52 (1H, dd, *J* = 12.1, 2.4 Hz, H-9_A_), 3.93 (1H, m, H-9_B_), 6.70 (2H, s, H-2′, 6′), 6.58 (1H, d, *J* = 15.9 Hz, H-7′), 6.31 (1H, dt, *J* = 15.9, 5.5 Hz, H-8′), 4.38 (2H, d, *J* = 5.5 Hz, H-9′).
